# Paraneoplastic necrotizing myopathy in a woman with breast cancer: a case report

**DOI:** 10.1186/1752-1947-3-95

**Published:** 2009-11-02

**Authors:** Joana Silvestre, Luis Santos, Vitor Batalha, Ana del Rio, Carlos Lima, Antonio Carvalho, Ana Martins, Helena Miranda, Fatima Cabral, Adelia Felix, Ana Aleixo

**Affiliations:** 1 Medicine IV Department, São Francisco Xavier Hospital, Estrada do Forte do Alto do Duque, 1449-005 Lisbon, Portugal

## Abstract

**Introduction:**

Paraneoplastic necrotizing myopathy is a rare disorder, described as a proximal, symmetrical, and rapidly progressing myopathy that is manifested as a paraneoplastic syndrome. Diagnosis is established via histological examination of the muscle biopsy.

**Case presentation:**

We present the case of a 53-year-old woman, born in Guinea-Bissau, with a history of locally advanced breast cancer, diagnosed ten months previously. The patient had experienced a progressively proximal muscle weakness of the lower extremities, which led to a total inability to walk. Upon neurological examination, the patient showed muscle weakness and atrophy in both proximal lower extremities without myalgia. Muscle strength was graded according to the Medical Research Council Scale as 2 out of 5 in the bilateral iliopsoas muscle, and 4 out of 5 in the bilateral quadriceps femoris. The deep-tendon reflexes were hypoactive. The laboratory examination showed increased values of serum creatinine kinase and myoglobin. An electromyogram showed an incomplete interference pattern during voluntary contraction in the iliopsoas and quadriceps femoris. The motor nerve conduction was 44.1 m/s and 44.3 m/s in the right and left tibial nerves, respectively, and 46.5 m/s and 46.1 m/s in the right and left peroneal nerves, respectively. The sensory motor nerve conductions and the compound motor action potential amplitudes were normal. These findings, despite not being specific, suggested a myopathy. Consequently, a muscle biopsy was performed. A biopsy specimen showed myopathic changes that were characteristic of a necrotizing myopathy.

**Conclusion:**

Treatment for this syndrome consists of controlling the tumor, and providing corticoid therapy. This led to the partial remission of the neurological manifestations.

## Introduction

Neurologic paraneoplastic syndromes are characterized by neurological dysfunctions occuring in patients with cancer that are not attributed to a direct invasion of the central nervous system or to metastatic lesions. Neurologic paraneoplastic syndromes occur in less than 1% of patients with cancer [1] and usually precede the diagnosis of the cancer. The etiopathogenesis of these entities is unknown, and they are classified according to their anatomic location [2,3].

Paraneoplastic necrotizing myopathy is a rare entity. In the published literature, Levin *et al. *were only able to identify four patients. Necrotizing myopathy has been associated with multiple neoplasms, the most frequent being gastrointestinal tract adenocarcinoma, prostate adenocarcinoma, bladder cancer, and non-small cell lung cancer [4,5].

This entity is characterized by a symmetric and proximal myopathy accompanied by an increase of the serum levels of the muscle enzymes and by electromyography (EMG) findings suggestive of a nonspecific myopathy [2,6]. The diagnosis of paraneoplastic necrotizing myopathy is histological [5]. The muscle biopsy shows evidence of a massive necrosis of the muscle fibers with almost complete absence of inflammatory infiltrate. This latter feature allows necrotizing myopathy to be distinguished from other myopathies also associated with paraneoplastic neurologic syndromes, such as dermatomyositis and polymyositis [7,8].

We present the case of a female patient with a locally advanced breast carcinoma, and with a final diagnosis of necrotizing myopathy. The diagnostic steps and subsequent therapeutic attitudes are discussed.

## Case presentation

A 53-year-old woman, born in Guinea-Bissau, was admitted in São Francisco Xavier Hospital with a progressively proximal muscle weakness of the lower extremities that had led to a total inability to walk. The patient had a history of a locally advanced breast cancer diagnosed ten months earlier. The tumour was 4 × 5 cm and located in her right breast with ulceration to the skin and with axillary adenopathies. Biopsies revealed an invasive ductal carcinoma with extensive tumor necrosis. Immunocytochemical staining was negative for hormonal receptors (estrogens and progesterone) and positive for c-erbB2 (Score 3+). The staging of the neoplastic disease, conducted through axillary echography, computerized tomography (CT) of the thorax, abdomen and pelvis, and bone scintigraphy, revealed a IIIC stage according to the Tumor-Node-Metastases (TNM) classification. Primary chemotherapy was performed with epirubicin and docetaxel, associated with radiotherapy of the primary lesion (3400 rads total). Given the absence of a reduction of the tumoral mass, the chemotherapy scheme was modified to cyclophosphamide, methotrexate and 5-fluorouracil (CMF).

Ten months later, the patient presented with signs of lower back pain with radiation towards both lower extremities accompanied by a decrease in muscle strength. Upon neurological examination, the patient presented muscle weakness and atrophy of both proximal lower extremities without myalgia. Muscle strength was graded according to the Medical Research Council Scale (MRC) as 2 out of 5 in the bilateral iliopsoas muscle, and 4 out of 5 in the bilateral quadriceps femoris. The deep-tendon reflexes were hypoactive. No other findings were encountered in the neurological examination.

There was no history of diabetes mellitus, alcoholism, toxin exposure, blood transfusion, nutritional deficiency, or any medication besides the chemotherapy above mentioned.

The following complementary examinations were performed: lumbar puncture, CT and magnetic resonance scans of the lumbosacral column, and tumor markers. All the results were normal. The laboratorial evaluation at this time indicated only a small elevation of creatinine kinase (CK) values (300 IU/l). In view of these results, the provisional diagnosis of secondary neuropathy due to docetaxel was considered, with the patient maintaining the CMF cycles and starting gabapentin. At this stage, and given the reduction of the tumor mass and partial recovery of the muscular strength (according to the MRC Scale as 4 out of 5 in the bilateral iliopsoas muscle, and 4 out of 5 in the bilateral quadriceps femoris), the patient was submitted to a modified radical mastectomy and was discharged from the hospital.

Three months later, the patient was readmitted due to new neurological deterioration. Muscle strength was graded according to the MRC Scale as 2 out of 5 in the bilateral iliopsoas muscle and 2 out of 5 in both quadriceps femoris, although it was nearly normal in other muscles. The muscles innervated by cranial nerves were spared. The deep-tendon reflexes were hypoactive.

The laboratorial re-evaluation showed a marked increase in serum CK values (2400 IU/l), serum myoglobin (780 IU/l), and moderate elevation of serum aminotransferases (alanine aminotransferase: 300 IU/l). Viral examination (serological markers for hepatitis B, C, HIV and human T-lymphotropic virus) was negative.

An EMG showed an incomplete interference pattern with poor activation during voluntary contraction in the iliopsoas and quadriceps femoris. The motor nerve conduction was 44.1 m/s and 44.3 m/s in the right and left tibial nerves, respectively, and 46.5 m/s and 46.1 m/s in the right and left peroneal nerves, respectively. The sensory motor nerve conductions and the compound motor action potential amplitudes were normal.

**Figure 1:**
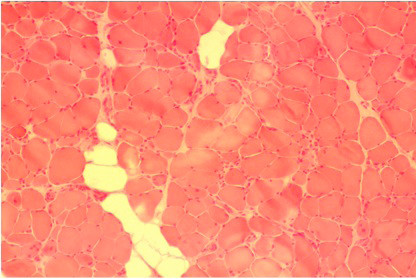
**Muscle biopsy of the quadriceps femoris (hematoxilin-eosin stain, ×100)**. Necrotic vacuolated and regenerating muscle fibres are present. Endomysial connective tissue is increased. Inflammatory infiltrate is absent.

**Figure 2:**
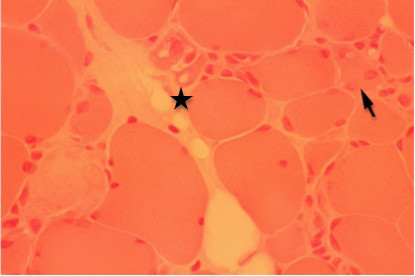
**Detail of muscle biopsy of the quadriceps femoris (hematoxilin-eosin stain, ×350), showing myopathic changes composed of scattered necrotic (star) and regenerating (arrow) fibers**.

**Figure 3:**
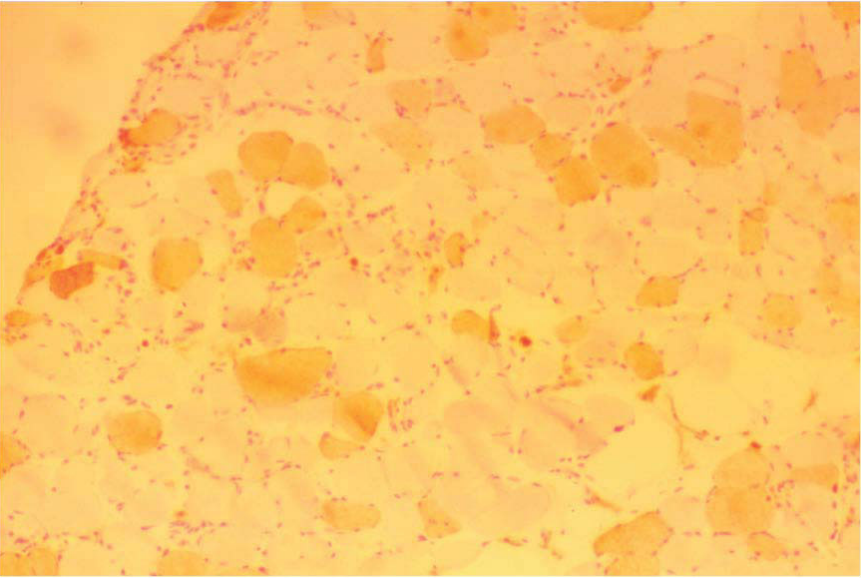
**Muscle biopsy of the quadriceps femoris (alkaline phosphates stain, ×100)**. This staining emphasizes the regenerating fibers and perimysial connective tissue.

**Figure 4:**
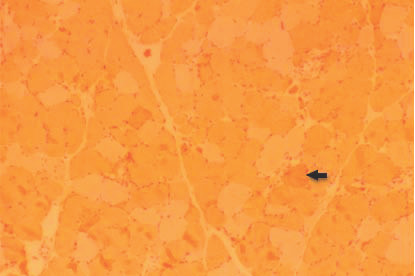
**Muscle biopsy of the quadriceps femoris (alkaline phosphates stain, ×100) showing myopathic changes composed of scattered necrotic (arrow) fibers**.

One week later, a biopsy specimen of the left quadriceps femoris presented a muscle necrosis pattern, increased centrally placed nuclei consisting of 3% of total fibers, and substantial numbers of scattered necrotic and regenerating fibers without inflammatory cells. A diffuse replacement of the muscle tissue by adipose and conjunctive tissues was also encountered (Figure 1, Figure 2, Figure 3 and Figure 4).

The following screening tests for inflammatory myopathies were conducted: antinuclear antibodies; anti-smooth muscle antibodies (ab); anti-ribonucleoprotein ab; anti-proliferating cell nuclear antigen ab; anti Mi2 ab; anti Jo1 ab; anti-SS A and anti SS B ab; and anti-scl 70 ab. Complement levels were normal.

A new staging of the neoplastic disease was also conducted, and revealed an absence of metastases.

The diagnosis of necrotizing myopathy was attributed, and corticotherapy was initiated (prednisolone 1.5 mg/kg/day), while still maintaining the chemotherapy already established. A full recovery of muscle strength was observed.

Four months later, a new decrease of muscle strength was observed, and the patient noticed difficulties standing up and raising her arms above shoulder height. Muscle strength was graded according to MRC as 3 out of 5 in the bilateral deltoids, biceps brachii and triceps brachii, and 2 out of 5 in the bilateral iliopsoas muscle and in both quadriceps femoris, but still maintaining distal strength. The muscles innervated by cranial nerves were still spared.

The EMG and muscle biopsy of the left biceps brachii muscle were repeated one week later. The EMG showed the same incomplete interference pattern during voluntary contraction in bilateral deltoids, biceps brachii, triceps brachii, iliopsoas and quadriceps femoris. The motor nerve conduction velocity was: 44.4 and 45 m/s in right and left median nerves, 49.5 and 48.7 m/s in right and left ulnar nerves, and 39.6 and 40.3 m/s in right and left peroneal nerves, respectively. The sensory motor nerve conductions and the compound motor action potential amplitudes were still normal. The biopsy specimen showed the same myopathic changes encountered in the first muscle biopsy.

At the same time, the re-staging of the neoplastic disease showed evidence of bone metastases in the sternum, which were confirmed by bone tomography and scintigraphy. Reinstitution of chemotherapy was established with epirubicin and docetaxel, and corticotherapy was intensified (3 mg/kg/day).

Three weeks later a progressive improvement of the muscle strength was observed with a partial recovery of the ability to walk. Muscle strength was graded according to MRC as 4 out of 5 in bilateral deltoids, biceps brachii, and triceps brachii and 3 out of 5 in the bilateral iliopsoas muscle and in both quadriceps femoris. The serum muscle enzymes returned to normal values.

The patient died one year later with pulmonary and hepatic failure due to metastasization of the underlying cancer.

## Discussion

In this report we describe a paraneoplastic necrotizing myopathy in a patient with breast cancer. The patient presented a progressive, proximal and symmetric myopathy with serum increases of the muscle enzymes. These elements should point to this entity. Despite these findings, diagnosis is only established through muscle biopsy. The histological examination of the biopsy revealed a massive necrosis of muscle fibers without inflammatory cell infiltrates. This latter feature allows the pathologist to distinguish necrotizing myopathy from other myopathies that are frequently associated to paraneoplastic syndromes, such as dermatomyositis and polymyositis.

In our patient, however, the diagnostic strength of the muscle biopsy had some limitations due to lack of immunohistochemical stainings for inflammatory cells and complement. Nevertheless, the results observed (a massive necrosis of muscle fibers without inflammatory T-cells infiltrates) in the hematoxylin-eosin and in acid phosphatase stainings support the diagnosis of a paraneoplastic necrotizing myopathy.

Paraneoplastic necrotizing myopathy associated with breast cancer is a rare entity: only three cases have been described in literature [5]. The absence of inflammation suggests that humoral immunologic mechanisms may be involved [5].

The rapid and symmetrical progression of this neurological condition suggests a radicular lumbosacral syndrome due to meningeal carcinomatosis or medullar compression. Because they occur more often among cancer patients, these diagnoses could delay the diagnosis of necrotizing myopathy. These hypotheses were therefore excluded.

The initial hypothesis of secondary neuropathy due to toxins or drugs was also excluded since there was no history of toxic exposure and no recovery was observed when chemotherapy with docetaxel was modified. Despite these drugs usually causing sensitive neuropathy, severe sensorimotor neuropathy has been described in some patients [2,9]. For this patient, however, the clinical picture (proximal weakness without sensory symptoms) was more consistent with a diagnosis of myopathy. This diagnosis was also supported by the findings in the EMG.

The aggressiveness of the underlying neoplastic disease, demonstrated by a difficult response to antineoplastic therapy, could lead to difficulty in improving neurological symptoms. Because paraneoplastic syndromes differ widely from one patient to another, prognosis may vary greatly according to the clinical and immunological features. Two groups of disorders have been considered: one group includes disorders mediated by antibodies against cell surface neuronal antigens - these syndromes often respond to treatment and have a favorable outcome, and another group includes disorders mediated by T-cell mechanisms, which respond poorly to treatment, and have a less favourable outcome [10].

There could, therefore, be a parallel between the severity of the underlying neoplastic disease and the clinical severity of the paraneoplastic syndrome. This patient showed improvements, with a partial recovery of the ability to walk, when the immunosuppressive therapy was intensified.

## Conclusion

Paraneoplastic syndromes are rare neurological complications in patients with cancer [11]. Necrotizing myopathy can induce a worse prognosis and a greater disability for the patient. It is important, therefore, to establish an early diagnosis of this entity because prompt diagnosis of the tumor and immunosuppressive therapy may positively impact the clinical outcome and improve the quality of life of patients with paraneoplastic syndromes.

## Abbreviations

Ab: antibodies; CK: creatinine kinase; CMF: cyclophosphamide, methotrexate and 5-fluorouracil; CT: computerized tomography; EMG: electromyogram; MRC: Medical Research Council Scale; TNM: Tumor-Node-Metastases.

## Consent

Written informed consent was obtained from the patient's family for publication of this case report and any accompanying images. A copy of the written consent is available for review by the Editor-in-Chief of this journal.

## Competing interests

The authors declare that they have no competing interests and confirm that all authors have seen and agree with the contents of the manuscript and agree that the work has not been submitted or published elsewhere in whole or in part.

## Authors' contributions

JS, LS, AC, VB, FC, AA analyzed and interpreted the patient's data regarding the neurological disease. AM, ADR, HM were responsible for the chemotherapy. LS performed the muscle biopsy and the EMG. CL performed the histological examination of the muscle biopsy. JS was involved in writing the manuscript. VB participated in the preparation of the manuscript and revised the article for intellectual content details. All authors read and approved the final manuscript.

## References

[B1] Darnell RB Posner JB Paraneoplastic syndromes involving the nervous system N Engl J Med 2003 349 16 1543 1554 10.1056/NEJMra023009 14561798

[B2] Arnold SM Lowy AM Patchell R Foon KA DeVita V, Hellman S, Rosenberg S Paraneoplastic syndromes Cancer. Principles and Practice of Oncology 2001 6 Philadelphia: Lippincott Williams & Wilkins 2511 2538

[B3] Mares-Segura R Spinal cord paraneoplastic syndromes Rev Neurol 2000 31 12 1219 1223 11205563

[B4] Emslie-Smith AM Engel AG Necrotizing myopathy with pipestem capillaries, microvascular deposition of the complement membrane attack complex (MAC), and minimal cellular infiltration Neurology 1991 41 6 936 939 2046947 10.1212/wnl.41.6.936

[B5] Levin MI Mozaffar T Al-Lozi MT Pestronk A Paraneoplastic necrotizing myopathy: clinical and pathological features Neurology 1998 50 3 764 767 9521271 10.1212/wnl.50.3.764

[B6] Dalmau J Rosenfeld MR Kasper DL, Braunwald E, Fauci A, Hauser S, Longo D, Jameson JL Paraneoplastic neurologic syndromes Harrison's Principles of Internal Medicine 2005 16 New York: McGraw-Hill 571 575

[B7] Hilton-Jones D Inflammatory muscle diseases Curr Opin Neurol 2001 14 5 591 596 10.1097/00019052-200110000-00007 11562570

[B8] Nanni C Fanti S Pinto C Farsad M Moretti A Franchi R Martoni A Monetti N Scintigraphic findings in necrotizing myopathy Clin Nucl Med 2003 28 2 118 120 10.1097/00003072-200302000-00005 12544128

[B9] Fazio R Quattrini A Bolognesi A Bordogna G Villa E Previtali S Canal N Nemni R Docetaxel neuropathy: a distal axonopathy Acta Neuropathol 1999 98 6 651 653 10.1007/s004010051132 10603043

[B10] Gallego Perez-Larraya J Dalmau J Classic paraneoplastic syndromes: diagnostic and treatment approach Neurologia 2008 23 7 441 448 18726722

[B11] Gatti G Simsek S Kurne A Zurrida S Naninato P Veronesi P Frasson A Millen E Rososchansky J Luini A Paraneoplastic neurological disorders in breast cancer Breast 2003 12 3 203 207 10.1016/S0960-9776(03)00011-0 14659327

